# Provider-generated barriers to health services access and quality still persist

**DOI:** 10.9745/GHSP-D-13-00162

**Published:** 2013-11-14

## Abstract

Barriers to access and quality, such as long waits, disrespectful provider behavior, and medical barriers, continue to constrain health programs. Reducing them further requires a multipronged management approach that includes understanding and addressing provider behavior and the real problems providers face.

Barriers to access and to the closely related issues of quality have been long recognized as important health program constraints.^1^ Such barriers have been addressed by a number of initiatives, including by improving guidelines, standards, and norms, and by various quality improvement approaches. Two articles in this issue of GHSP—Tumlinson et al. for family planning and Amin et al. for immunization—examine this issue using a variety of methodologies including mystery clients. Unfortunately, important provider-generated barriers are still alive and well.

Among the most prominent barriers:

- Disrespect and verbal abuse
- Lack of service—for example, provider absences, service not provided that day
- Medical barriers—such as requiring women to be menstruating in order to obtain a family planning method and not providing immunizations if a child has an illness
- Inadequate counseling
- Inappropriate fees (Tumlinson only)

The first of these—disrespect and abuse—has recently emerged as a major concern in the maternal health area.^2^ Rudeness and abuse take on a particularly poignant role in that arena, because women in labor and at delivery are vulnerable and, in a sense, captive. Notably, Amin et al. found that previous provider rudeness in maternity care appeared to spill over to discourage caregivers from seeking immunization for their children.

On the positive side, much of what providers are doing appears to demonstrate reasonably good access and quality. So what will it take to reduce the important barriers that persist? There is, of course, no single answer. But it does require awareness of the problem and serious approaches to remedy it, including: training, supervision, management, further technical policy change, better supply chain, job aids, and engaging the consumer community to demand consistent and respectful service.

However, we also need to empathize with providers and get a better grasp of their perspective. Amin et al. give some insight on the provider prospective, including heavy workloads, lack of staff, requirement to provide multiple services (multitasking), and lack of transport.

These should come as no surprise. We know many such problems are common in health systems. Improving them, and improving access and quality, has no single solution but requires good systems and management thinking to address the problems. The worst thing we can do is pretend they do not exist. –*Global Health: Science and Practice*
